# Post-transplant cyclophosphamide containing regimens after matched sibling, matched unrelated and haploidentical donor transplants in patients with acute lymphoblastic leukemia in first complete remission, a comparative study of the ALWP of the EBMT

**DOI:** 10.1186/s13045-021-01094-2

**Published:** 2021-05-28

**Authors:** Jaime Sanz, Jacques-Emmanuel Galimard, Myriam Labopin, Boris Afanasyev, Moiseev Ivan Sergeevich, Emanuele Angelucci, Nicolaus Kröger, Yener Koc, Fabio Ciceri, J. L. Diez-Martin, Mutlu Arat, Simona Sica, Montserrat Rovira, Mahmoud Aljurf, Johanna Tischer, Bipin Savani, Annalisa Ruggeri, Arnon Nagler, Mohamad Mohty

**Affiliations:** 1 Hematology Department, Hospital Universitari i Politècnic La Fe, Valencia, Spain; 2 CIBERONC, Instituto Carlos III, Madrid, Spain; 3 EBMT Paris Study Office, Department of Haematology, Saint Antoine Hospital, INSERM UMR 938, Sorbonne University, Paris, France; 4 RM Gorbacheva Research Institute, Pavlov University, Lva Tolstogo 6/8, 197022 Saint-Petersburg, Russian Federation; 5 Hematology and Transplant Center, IRCCS Ospedale Policlinico San Martino, Genova, Italy; 6 Bone Marrow Transplantation Centre, University Hospital Eppendorf, Hamburg, Germany; 7 Medicana International, Istanbul, Turkey; 8 Ospedale San Raffaele s.r.l., Haematology and BMT, Milan, Italy; 9 Hematology Department, Hospital GU Gregorio Marañon, Instituto de Investigación Sanitaria Gregorio Marañon, Universidad Complutense Madrid, Madrid, Spain; 10 Florence Nightingale Sisli Hospital, Hematopoietic SCT Unit, Istanbul, Turkey; 11 Istituto di Ematologia, Universita Cattolica S. Cuore, Rome, Italy; 12 Department of Hematology, Hospital Clinic, Institute of Hematology and Oncology, Barcelona, Spain; 13 August Pi I Sunyer (IDIBAPS), University of Barcelona, Barcelona, Spain; 14 King Faisal Specialist Hospital and Research Centre Oncology (Section of Adult Haematolgy/BMT), Riyadh, Saudi Arabia; 15 Department of Internal Medicine III, Grosshadern, LMU, University Hospital of Munich, Munich, Germany; 16 Vanderbilt University Medical Center, Nashville, TN USA; 17 Division of Hematology and Bone Marrow Transplantation, The Chaim Sheba Medical Center, Tel-Hashomer, Ramat-Gan, Israel; 18 Department of Hematology, and INSERM UMRs 938, Hopital Saint Antoine, Sorbonne University, Paris, France

**Keywords:** Post-transplant cyclophosphamide, Haploidentical transplant, Alternative donor transplants, Acute lymphoblastic leukemia, Allogeneic stem cell transplant

## Abstract

**Background:**

There is no information on the impact of donor type in allogeneic hematopoietic stem cell transplantation (HCT) using homogeneous graft-versus-host (GVHD) prophylaxis with post-transplant cyclophosphamide (PTCy) in acute lymphoblastic leukemia (ALL).

**Methods:**

We retrospectively analyzed outcomes of adult patients with ALL in CR1 that had received HCT with PTCy as GVHD prophylaxis from HLA-matched sibling (MSD) (*n* = 78), matched unrelated (MUD) (*n* = 94) and haploidentical family (Haplo) (*n* = 297) donors registered in the EBMT database between 2010 and 2018. The median follow-up period of the entire cohort was 2.2 years.

**Results:**

Median age of patients was 38 years (range 18–76). Compared to MSD and MUD, Haplo patients received peripheral blood less frequently. For Haplo, MUD, and MSD, the cumulative incidence of 100-day acute GVHD grade II–IV and III–IV, and 2-year chronic and extensive chronic GVHD were 32%, 41%, and 34% (*p* = 0.4); 13%, 15%, and 15% (*p* = 0.8); 35%, 50%, and 42% (*p* = 0.01); and 11%, 17%, and 21% (*p* = 0.2), respectively. At 2 years, the cumulative incidence of relapse and non-relapse mortality was 20%, 20%, and 28% (*p* = 0.8); and 21%, 18%, and 21% (*p* = 0.8) for Haplo, MUD, and MSD, respectively. The leukemia-free survival, overall survival and GVHD-free, relapse-free survival for Haplo, MUD, and MSD was 59%, 62%, and 51% (*p* = 0.8); 66%, 69%, and 62% (*p* = 0.8); and 46%, 44%, and 35% (*p* = 0.9), respectively. On multivariable analysis, transplant outcomes did not differ significantly between donor types. TBI-based conditioning was associated with better LFS.

**Conclusions:**

Donor type did not significantly affect transplant outcome in patient with ALL receiving SCT with PTCy.

## Introduction

The role of allogeneic hematopoietic stem cell transplantation (alloHCT) in adults with acute lymphoblastic leukemia (ALL) has recently been critically evaluated in two systematic evidence-based reviews, one by the American Society for Transplantation and Cellular Therapy (ASTCT) [1] and the other by the European Working Group for Adult Acute Lymphoblastic Leukemia (EWALL) and the Acute Leukemia Working Party (ALWP) of the European Society for Blood and Marrow Transplantation (EBMT) [2]. Both documents state that alloHCT is considered the best option for adult patients with ALL in first complete remission (CR1) with high-risk features. In addition, there was a consensus in considering that the preferred donor in this setting is an HLA-matched sibling donor (MSD) or matched unrelated donor (MUD). For patients with ALL in need of alloHCT lacking an HLA-matched donor, alternative donor options such as a haploidentical family donor (Haplo), mismatched unrelated donor (MMUD), and unrelated umbilical cord blood transplantation can be considered [3–6].

A few recent studies of adult patients with high-risk ALL undergoing haploidentical HCT have reported favorable transplant outcomes, which has led to the consideration of this approach as a valid option for patients lacking a matched donor [4, 5, 7–10]. In patients with this disease undergoing haploidentical HCT, post-transplant cyclophosphamide (PTCy), in the absence of prospective randomized data, may be considered over ATG [10]. There has been increased interest of comparing haploidentical HCT with other stem cell donors. Indeed, two recent studies of the ALWP of the EBMT comparing transplant outcomes in patients with ALL who underwent haploidentical and MUD transplant showed comparable results [4, 5]. One of them analyzed patients in CR1 using PTCy- and ATG-based GVHD prophylaxis [5], while the other is a joint study of the EBMT with the Transplant and Cellular Therapy–Research Consortium (TCT-RC) [4], in which they performed a matched-pair comparison in patients who underwent haploidentical HCT with PTCy-based GVHD prophylaxis and MUD transplant [5]. In a latest multicenter biologically phase III randomized study, Wang et al. [7] showed comparable outcomes in patients with ALL in CR1 who received alloHCT from MSD or haploidentical donor using an ATG-based strategy. Very recently we compared haploidentical HCT to alloHCST from MSD in patients with ALL in CR demonstrating higher relapse rates while lower transplant-related mortality in the MSD transplants [11]. However, as far as we know, none of these studies compared outcomes of haploidentical HCT with both alloHCT from MSD or MUD using a homogeneous PTCy-based GVHD prophylaxis in patients with ALL in CR1.

The aim of this study was to investigate the impact of donor type on the outcome of patients with ALL in CR1 undergoing unmanipulated alloHCT using PTCy as GvHD prophylaxis. We analyzed patients who had received alloHCT from MSD, MUD and Haplo family donors, reported to the EBMT registry between 2010 and 2018.

## Patients and methods

### Study design and data source

This is a retrospective registry-based analysis on behalf of the Acute Leukemia Working Party (ALWP) of the EBMT. The EBMT is a voluntary working group of more than 600 transplantation centers that are required to report all consecutive stem cell transplantations and follow-up once a year. In EBMT registry, there is an internal quality control regarding accuracy and consistency of what is entered data and periodic queries on missing / incorrect data and follow-up requests. A routinely audit, however, will not be performed. All transplantation centers are required to obtain written informed consent before data registration with the EBMT in accordance with the 1975 Declaration of Helsinki.

### Patient eligibility

All adults (aged ≥ 18 years) with ALL in CR1 at transplantation, registered in the EBMT ProMISe database, who underwent a first alloHCT from an unmanipulated graft, using PTCy from Haplo, MUD or MSD donors between 2010 and 2018 were eligible. MUD was defined as 10/10 patient and donor compatibility considering HLA A, B, C, and DRB1 and DQB1 allelic typing. Haplo transplant was defined as one with the number of donor–recipient human leukocyte antigen (HLA) mismatches ≥ 2.

### Endpoints and definitions

The primary endpoint was leukemia-free survival (LFS) after MSD, MUD, and Haplo donor transplants. Secondary endpoints were neutrophil engraftment, acute GVHD (aGVHD) and chronic GVHD (cGVHD), relapse incidence, non-relapse mortality (NRM), GVHD-free, relapse-free survival (GRFS), and overall survival (OS) within the same subgroups and to perform analysis of risk factors for each outcome.

Neutrophil recovery was defined as the first day of an absolute neutrophil count of 0.5 × 10^9^/L lasting for ≥ 3 consecutive days. Acute GVHD and cGVHD were defined and graded according to standard criteria [12, 13]. Relapse was defined as disease recurrence and appearance of blasts in the peripheral blood (PB) or bone marrow (> 5%) after CR. LFS was calculated until the date of first relapse, death from any cause, or the last follow-up. NRM was defined as death from any cause other than relapse. The composite endpoint GRFS was defined as survival without the following events: stage III–IV aGVHD, severe cGVHD, disease relapse, or death from any cause after SCT [14]. Myeloablative conditioning (MAC) was defined as a regimen containing either total body irradiation (TBI) with a dose > 6 Gray, a total dose of oral busulfan > 8 mg/kg, or a total dose of intravenous busulfan > 6.4 mg/kg [15]. All other regimen intensities were defined as reported by the centers.

### Statistical analysis

Patient characteristics according to donor type were compared using the chi-square test for categorical and Kruskal-Wallis test for continuous variables. GRFS, LFS, and OS were estimated using the Kaplan–Meier method. Cumulative incidence functions were used to estimate neutrophil engraftment, aGVHD, cGVHD, relapse incidence, and NRM. Competing risks were death for relapse incidence and neutrophil engraftment, relapse for NRM, relapse or death for aGVHD and cGVHD. Univariate analyses were performed using the log-rank test for LFS, GRFS, and OS, and Gray’s test for cumulative incidence. Multivariate analyses were performed using the Cox proportional-hazards model [16].

Donor type, gender, age at transplantation, Karnofsky performance status, transplantation year, patient cytomegalovirus serostatus, use of TBI, and type of ALL (using the Phi positivity) were included in the final model. The missing data on Ph positivity were handled as a supplementary category in multivariate models for adjustment. Stem cell source and use of ATG were not included in the model due to their association with donor type. TBI was used over conditioning intensity due to low numbers of TBI-based RIC in MSD (*n* = 13) and MUD (*n* = 12). To allow the center effect, we introduced a random effect (frailty term) for each center into the model. Median follow-up was estimated using the reverse Kaplan–Meier method. The significance level was fixed at 0.05, and P values were two-sided. Statistical analyses were performed using R software version 4.0.2 (R Development Core Team, Vienna, Austria) software packages.

## Results

### Patient and transplantation characteristics

Patient, disease, and transplant characteristics of the overall study population and according to donor type are summarized in Table 1. A total of 469 patients were included in the study, of which 297 were transplanted from Haplo, 94 from MUD and 78 from MSD donors. The median age of patients was 37 years (range 18–76) and 64% were male. ALL was of B-cell origin in 359 (77%) patients and 166 (41%) were Ph positive, 243 (59%) Ph negative (including all T-cell types) and the Ph data was missing for 60 patients. PB was used as the stem cell source in 293 (62%) patients. Regarding conditioning, 209 (45%) were TBI-based regimens and 387 (83%) patients received MAC. PTCy was used alone in (*n* = 27; 6%) or in combination with 1 (*n* = 75; 16%) or 2 (*n* = 367; 78%) immunosuppressive drugs. Most frequent immunosuppressive drugs associated with PTCy were: combination of mycophenolate mofetil and calcineurin inhibitors (*n* = 318; 68%), calcineurin inhibitors alone (*n* = 44; 9%), methotrexate with (*n* = 29; 6%) or without cyclosporine (*n* = 17; 4%), and combination of mycophenolate mofetil and sirolimus (*n* = 14; 3%). In vivo T-cell depletion (TCD), mainly ATG, was used in 58 (12%) patients, 23 (8%) in Haplo, 13 (17%) in MSD and 22 (23%) in MUD.

**Table 1:** Patient, disease, and transplant characteristics according to donor type

Characteristics	Total N = 469	MSD N = 78	MUD N = 94	Haplo N = 297	*p*
Age in years, median (range)	37 (18–75)	37 (18–67)	33 (18–75)	39 (18–75)	0.2
Gender, *n* (%)					1
Male	299 (64)	50 (64)	60 (65)	189 (64)	
Female	170 (36)	28 (36)	34 (35)	108 (36)	
Karnofsky performance status, *n* (%)					0.4
≥ 90	352 (78)	53 (73)	67 (76)	232 (80)	
< 90	99 (22)	20 (27)	21 (24)	58 (20)	
Missing	18	5	6	7	
Cell lineage, *n* (%)					0.7
B-cell	359 (77)	57 (73)	73 (78)	229 (77)	
T-cell	110 (23)	21 (27)	21 (22)	68 (23)	
Ph-chromosome status, *n* (%)					
Negative	243 (59)	43 (64)	53 (61)	147 (58)	0.6
Positive	166 (41)	24 (36)	34 (39)	108 (42)	
Missing	60	11	7	42	
Months from diagnosis to transplant, median (range)	6 (1–18)	6 (1–18)	7 (3–18)	6 (1–18)	0.2
Conditioning intensity, *n* (%)					0.5
Myeloablative	387 (83)	63 (83)	82 (83)	242 (82)	
Reduced intensity	79 (17)	13 (17)	12 (17)	54 (18)	
Missing	3	2	0	1	
Type of conditioning, *n* (%)					0.6
Based on chemotherapy	259 (55)	40 (52)	56 (60)	163 (55)	
Based on TBI	209 (45)	37 (48)	36 (40)	134 (45)	
Missing	1	1	0	0	
Stem cell source, *n* (%)					< 0.001
Bone marrow	176 (38)	20 (26)	11 (12)	145 (49)	
Mobilized peripheral blood	293 (62)	58 (74)	83 (88)	152 (51)	
In vivo T-cell depletion, *n* (%)	58 (12)	13 (17)	22 (23)	23 (8)	< 0.001
GvHD prophylaxis, *n* (%)					< 0.001
PTCy + 2 drugs	367 (78)	25 (32)	69 (73)	273 (92)	
PTCy + 1 drug	75 (16)	32 (41)	21 (22)	22 (7)	
PTCy only	27 (6)	21 (27)	4 (4)	2 (1)	
Donor–recipient gender combination, *n* (%)					0.08
Female donor to male recipient	118 (25)	27 (35)	19 (20)	72 (24)	
Other combinations	350 (75)	51 (65)	74 (80)	225 (76)	
Missing	1	0	1	0	
Donor–recipient CMV serostatus, *n* (%)					< 0.001
Negative–negative	63 (14)	10 (14)	23 (26)	30 (11)	
Positive–negative	46 (10)	11 (15)	11 (12)	24 (8)	
Negative–positive	65 (15)	6 (8)	23 (26)	36 (13)	
Positive–positive	275 (61)	46 (63)	33 (37)	196 (69)	
Missing	20	5	4	11	
Year of transplant, median (range)	2016 (2010–2018)	2016 (2010–2018)	2016 (2010–2018)	2016 (2011–2018)	0.8

MSD, matched sibling donor; MUD, matched unrelated donor; Haplo, haploidentical donor; ALL, acute lymphoblastic leukemia; Ph, Philadelphia chromosome; TBI, total body irradiation; GvHD, graft-versus-host disease; PTCy, post-transplant cyclophosphamide; CMV, cytomegalovirus; *p*, *p* value

MSD, MUD, and Haplo recipients did not differ with respect to patient and disease characteristics. Regarding transplant characteristics, Haplo patients less frequently received TCD (*p* < 0.001) and PB (*p* < 0.001). Although most (92%) Haplo patients received GvHD prophylaxis with PTCy combined with two other immunosuppressive drugs, only 73% and 32% of MUD and MSD patients, respectively, received such a combination (*p* < 0.001).

Median follow-up was 2.2 years (95% CI 2–2.8) for the entire cohort, 2.6 years (95% CI 2.1–3) for Haplo, 1.9 years (95% CI 1.2–2.8) for MUD, and 2 years (95% CI 1.4–3) for MSD patients.

### Engraftment, acute and chronic GVHD

The cumulative incidence of neutrophil recovery at 60 days was 97% (95% CI 94–98) for Haplo, 100% for MUD, and 99% (95% CI 85–100) for MSD (*p* = 0.23). The median time to neutrophil recovery was the same at 18 days (interquartile range [IQR] 15–20), 18 days (IQR 14–21), and 18 days (IQR 14–20) for Haplo, MUD and MSD, respectively.

The cumulative incidence of aGvHD grade II–IV at 100 days was 32% (95% CI 27–38) for Haplo, 41% (95% CI 30–51) for MUD, and 34% (95% CI 23–45) for MSD (*p* = 0.4). The 100-day cumulative incidence of aGVHD grade III–IV was 13% (95% CI 9–17) for Haplo, 15% (95% CI 9–24) for MUD, and 15% (95% CI 8–24) for MSD (*p* = 0.8) (Table 2). On multivariable analysis, no variables were found to have a significant impact on the risk of aGvHD (Tables 3 and 4).

**Table 2:** Univariate analysis of transplant outcomes according to donor type

Outcome^a^	MSD	MUD	Haplo	*p*
Acute GvHD, % (95% CI)				
Grade II–IV	34 (23–45)	41 (30–51)	32 (27–38)	0.4
Grade III–IV	15 (8–24)	15 (9–24)	13 (9–17)	0.8
Chronic GvHD, % (95% CI)				
Overall	42 (29–54)	50 (37–61)	35 (29–41)	0.01
Extensive	21 (12–33)	17 (9–27)	11 (7–15)	0.2
NRM, % (95% CI)	21 (12–32)	18 (10–27)	21 (17–26)	0.8
RI, % (95% CI)	28 (17–41)	20 (12–30)	20 (16–26)	0.8
LFS, % (95% CI)	51 (37–63)	62 (50–72)	59 (52–64)	0.8
OS, % (95% CI)	62 (48–73)	69 (57–79)	66 (60–72)	0.8
GRFS, % (95% CI)	35 (23–47)	44 (32–55)	46 (40–52)	0.9

GvHD, graft-versus-host disease; CI, confidence interval; *p*, *p* value; NRM, non-relapse mortality; RI, relapse incidence; LFS, leukemia-free survival; OS, overall survival; GRFS, graft-versus-host disease-free, relapse-free survival; MSD, matched sibling donor; MUD, matched unrelated donor; Haplo, haploidentical donor

^a^Acute GvHD: 100-day cumulative incidence; cGvHD, NRM and RI: cumulative incidence at 2 years; DFS, OS and GRFS: survival probability at 2 years

**Table 3:** Multivariate analysis of transplant outcomes according to donor type

Outcome	MSD	MUD			Haplo		
	Reference	HR	95% CI	*p*	HR	95% CI	*p*
Acute GvHD grade II–IV	1	1.25	0.73–2.13	0.4	0.99	0.62–1.57	1
Overall chronic GvHD	1	1.84	1.02–3.34	0.04	1.03	0.59–1.79	0.9
NRM	1	1.06	0.47–2.38	0.9	1.16	0.60–2.25	0.7
RI	1	0.78	0.38–1.57	0.5	0.74	0.43–1.28	0.3
LFS	1	0.91	0.54–1.55	0.7	0.91	0.59–1.39	0.7
OS	1	1.05	0.57–1.94	0.9	1.13	0.68–1.86	0.6
GRFS	1	1.02	0.65–1.59	0.9	0.96	0.66–1.4	0.8

GvHD, graft-versus-host disease; HR, hazard ratio; CI, confidence interval; *p*, *p* value; NRM, non-relapse mortality; RI, relapse incidence; LFS, leukemia-free survival; OS, overall survival; GRFS, graft-versus-host disease-free, relapse-free survival; MSD, matched sibling donor; MUD, matched unrelated donor; Haplo, haploidentical donor

**Table 4:** Multivariate analysis of transplants outcomes

Variable	aGvHD II–IV		cGvHD		NRM		RI		LFS		OS		GRFS	
	HR (95% CI)	*p*	HR (95% CI)	*p*	HR (95% CI)	*p*	HR (95% CI)	*p*	HR (95% CI)	*p*	HR (95% CI)	*p*	HR (95% CI)	*p*
Donor type														
MSD	1		1		1		1		1		1		1	
MUD	1.25 (0.73–2.13)	0.42	**1.84 (1.02–3.34)**	**0.04**	1.06 (0.47–2.38)	0.89	0.78 (0.38–1.57)	0.48	0.91 (0.54–1.55)	0.74	1.05 (0.57–1.94)	0,.7	1.02 (0.65–1.59)	0,.3
Haplo	0.99 (0.62–1.57)	0.96	1.03 (0.59–1.79)	0.93	1.16 (0.6–2.25)	0.66	0.74 (0.43–1.28)	0.28	0.91 (0.59–1.39)	0.65	1.13 (0.68–1.86)	0.64	0.96 (0.66–1.4)	0.84
Patient’s age per 10 years^1^	1.01 (0.9–1.14)	0,83	1.03 (0.89–1.18)	0,72	**1.3 (1.1–1.55)**	**0,002**	0.95(0.81–1.11)	0,5	1.09 (0.97–1.22)	0,14	**1.15 (1.02–1.31)**	**0,03**	1.07 (0.97–1.18)	0,17
Patient’s gender														
Male	1		1		1		1		1		1		1	
Female	0.84 (0.59–1.19)	0.33	**0.59 (0.4–0.87)**	**0.008**	0.67 (0.41–1.09)	0.1	0.79 (0.5–1.24)	0.31	0.74 (0.53–1.03)	0.08	**0.68 (0.47–1)**	**0.04**	**0.75 (0.56–0.99)**	**0.04**
Karnofsky performance status														
Poor (< 90)	1		1		1		1		1		1		1	
Good (≥ 90)	1.12 (0.73–1.7)	0.61	0.65 (0.39–1.06)	0.09	0.78 (0.45–1.36)	0.55	1.2 (0.68–2.13)	0.53	0.94 (0.63–1.39)	0.75	0.82 (0.53–1.27)	0.38	1.03 (0.73–1.47))	0.86
Patient’s CMV serostatus														
Negative	1		1		1		1		1		1		1	
Positive	0.98 (0.66–1.44)	0.90	0.76 (0.5–1.17)	0.21	1.45 (0.78–2.71)	0.24	1.46 (0.85–2.52)	0.17	1.44 (0.95–2.17)	0.08	1.21 (0.77–1.91)	0.4	1.05 (0.75–1.46)	0.78
Cytogenetics														
Philadephia neg	1		1		1		1		1		1		1	
Philadephia pos	1.02 (0.7–1.47)	0.94	0.8 (0.53–1.21)	0.3	0.87 (0.52–1.43)	0.58	**0.54 (0.32–0.9)**	**0.02**	0.71 (0.5–1.02)	0.06	0.67 (0.45–1)	0.05	0.8 (0.59–1.09)	0.16
Philadephia unknown	0.75 (0.43–1.31))	0.32	1.06 (0.57–1.94)	0.86	1.07 (0.5–2.29))	0.87	0.80 (0.43–1.49)	0.48	0.89 (0.54–1.46)	0.64	0.86 (0.49–1.52)	0.61	0,78 (0.5–1.22)	0.27
Type of conditioning														
Based on chemotherapy	1		1		1		1		1		1		1	
Based on TBI	1.17 (0.85–1.63)	0.34	0.79 (0.52–1.2)	0,.7	0.68 (0.41–1.13)	0.13	0.68 (0.45–1.05)	0.08	**0.70 (0.51–0.98)**	**0.04**	0.78 (0.53–1.14)	0.20	0.78 (0.58–1.03)	0.08
Year of transplant^1^	0.98 (0.91–1.07)	0.69	**0.90 (0.81–0.99)**	**0.03**	0.93 (0.83–1.04)	0.21	0.95 (0.85–1.05)	0.28	0.93 (0.86–1)	0.07	**0.90 (0.82–0.98)**	**0.01**	0.97 (0.91–1.04)	0,47

Bold statistically significant variables for specific outcomes

1. Continuous variable

GvHD, graft-versus-host disease; CI, confidence interval; NRM, non-relapse mortality; RI, relapse incidence; LFS, leukemia-free survival; OS, overall survival; GRFS, graft-versus-host disease and relapse-free survival; TBI, total body irradiation

The 2-year cumulative incidence of cGVHD was 35% (95% CI 29–41), 50% (95% CI 37–61), and 42% (95% CI 29–54) (*p* = 0.01) for Haplo, MUD, and MSD, respectively (Table 2). The cumulative incidence of extensive cGVHD was 11% (95% CI 7–15) for Haplo, 17% (95% CI 9–27) for MUD, and 21% (95% CI 12–33) for MSD (*p* = 0.2) (Table 2). On multivariable analysis (Table 3), MUD showed an increased risk of cGVHD when compared with MSD (hazard ratio [HR] 1.84; 95% CI 1.02–3.34; *p* = 0.04). Other factors associated with a lower risk of cGVHD were female gender (HR 0.59; 95% CI 0.4–0.87; *p* = 0.008), and transplant performed more recently (HR 0.9; 95% CI 0.81–0.99; *p* = 0.03) (Table 4).

### Relapse

**Fig. 1:**
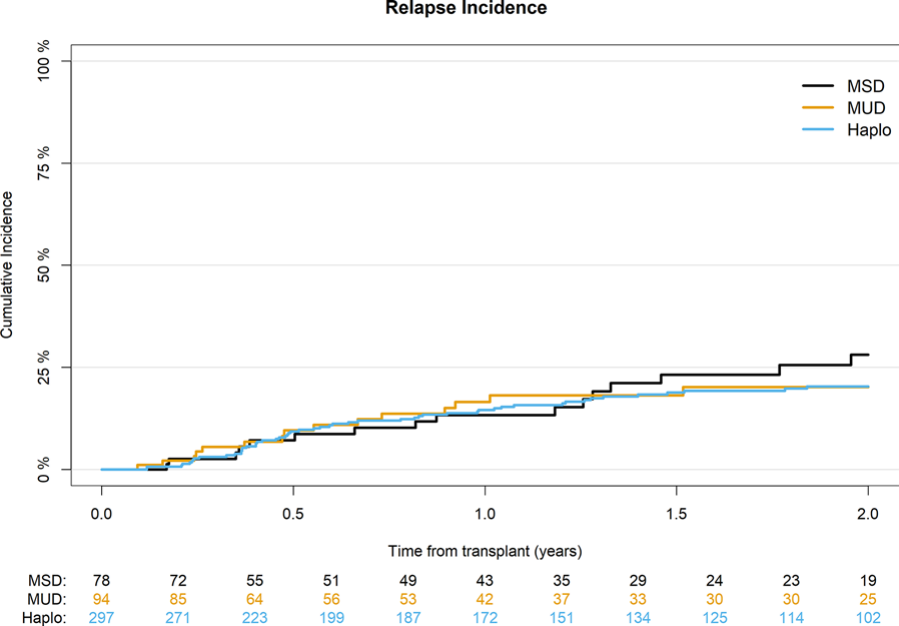
Cumulative incidence of relapse according to donor type

The median time to relapse was 7 months (IQR 4–14). The cumulative incidence of relapse at 2 years was not significantly different across the donor types with 20% (95% CI 16–26) for Haplo, 20% (95% CI 12–30) for MUD, and 28% (95% CI 17–41) for MSD (*p* = 0.8) (Table 2) (Fig. 1). On multivariable analysis, Ph + patients had a lower risk of relapse (HR 0.54; 95% CI 0.32–0.9; *p* = 0.02) (Table 4).

### NRM and causes of death

**Fig. 2:**
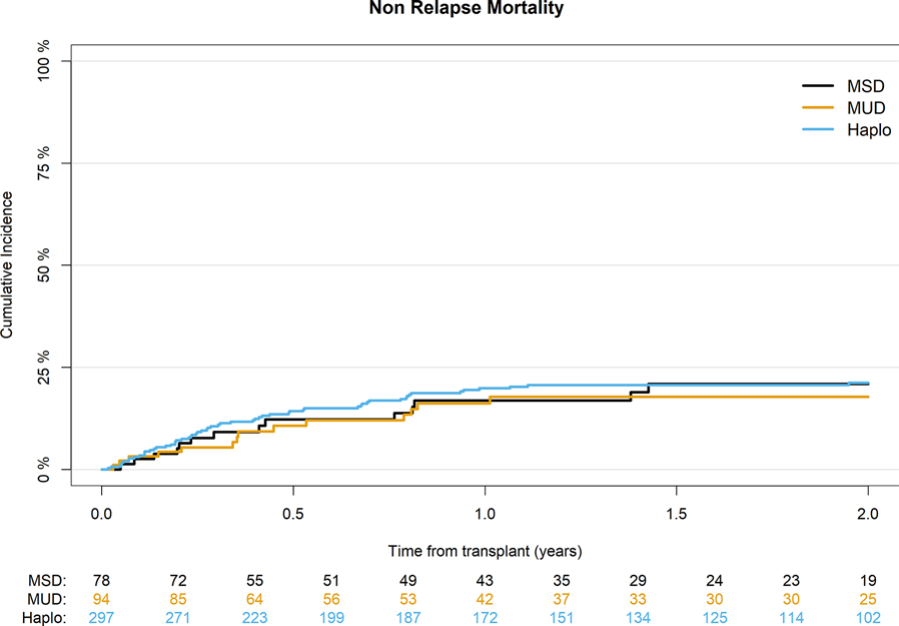
Cumulative incidence of non-relapse mortality according to donor type

The cumulative incidence of NRM at 2 years was 21% (95% CI 17–26) for Haplo, 18% (95% CI 10–27) for MUD, and 21% (95% CI 12–32) for MSD (*p* = 0.8) (Table 2) (Fig. 2). In multivariable analysis higher recipient’s age per 10 years (HR 1.3; 95% CI 1.1–1.55; *p* = 0.002) was the only factor significantly associated with an increased NRM (Table 4).

At last follow-up, 151 patients had died, of which 90 (60%) were due to a variety of non-relapse causes, 61 (60%) in Haplo, 15 (60%) in MUD, and 14 (58%) in MSD. The main causes of transplant-related deaths were infections and GvHD, being 25 (25%) and 16 (16%) in Haplo, 8 (32%) and 3 (12%) in MUD, and 4 (17%) and 6 (25%) in MSD cohorts, respectively (Table 5).

**Table 5:** Causes of death according to donor type

Causes of death	MSD *n* (%)	MUD *n* (%)	Haplo *n* (%)
Overall	24	25	102
Relapse	10 (42)	10 (40)	41 (40)
Infections	4 (17)	8 (32)	25 (25)
GvHD	6 (25)	3 (12)	16 (16)
Interstitial pneumonitis	2 (8)	3 (12)	4 (4)
Sinusoidal obstruction syndrome	0 (0)	0 (0)	2 (2)
Hemorrhage	0 (0)	0 (0)	1 (1)
Secondary malignancy	1 (4)	1 (4)	1 (1)
Graft failure	0 (0)	0 (0)	2 (2)
Other	1 (4)	0 (0)	10 (10)

### Survival outcomes

For the entire cohort, LFS, OS, and GRFS at 2 years were 58% (95% CI 53–63), 66% (95% CI 61–71), and 44% (95% CI 39–49), respectively.

**Fig. 3:**
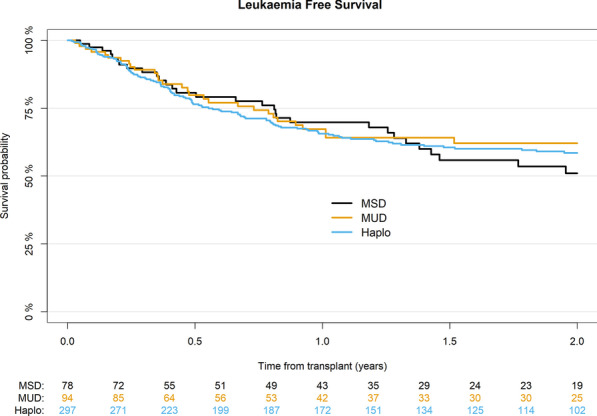
Probability of leukemia-free survival according to donor type

LFS was 59% for Haplo, 62% for MUD, and 51% for MSD (*p* = 0.8) (Table 2) (Fig. 3). On multivariate analysis, the use of TBI was associated with better LFS (HR 0.7; 95% CI 0.51–0.98; *p* = 0.04) (Table 4).

**Fig. 4:**
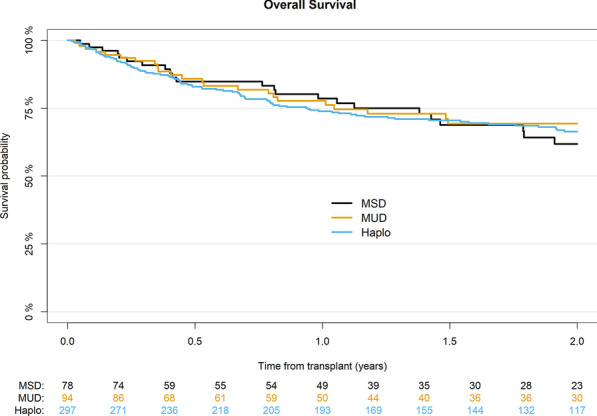
Probability of overall survival according to donor type

OS did not differ significantly with 66% for Haplo, 69% for MUD, and 62% for MSD (*p* = 0.8) (Table 2) (Fig. 4). On multivariate analysis, female gender (HR 0.68; 95% CI 0.47–1; *p* = 0.048) and transplants performed more recently (HR 0.9; 95% CI 0.82–0.98; *p* = 0.01) were associated with better OS, while higher recipient age per 10 years was associated with a worse OS (HR 1.15; 95% CI 1.02–1.31; *p* = 0.03) (Table 4).

GRFS was 46%, 44%, and 35% for Haplo, MUD, and MSD, respectively (*p* = 0.9) (Table 2). On multivariate analysis, female gender was associated with better GRFS (HR 0.75; 95% CI 0.56–0.99; *p* = 0.04) (Table 4).

## Discussion

This study shows that transplant outcomes in terms of NRM, relapse, and survival probabilities in patients with ALL in CR1 undergoing alloHCT were not significantly different according to donor type. This study provides evidence to support Haplo transplantation as a valuable alternative donor option in this setting when a matched donor is not available. Our results also show that the use of PTCy for GvHD prophylaxis in patients with ALL in CR1 receiving HCT from MSD, MUD and Haplo is safe and effective, resulting in low cumulative incidences of GVHD, especially cGVHD, in all transplant settings. Nevertheless, alloHCT from MUD had a significant increased risk of cGVHD than those from MSD and Haplo. In accordance to many previous publications TBI-based conditioning was associated with better LFS.

This study confirmed that PTCy was highly effective in preventing acute and chronic extensive GvHD in MSD, MUD, and Haplo HCT and seems to compare favorably with standard GvHD prophylaxis with a calcineurin inhibitor and methotrexate in MSD and MUD transplants [17, 18]. In fact, the incidence of GvHD after PTCy seems similar to that reported with ATG in both matched donor settings [19, 20]. The incidence of severe aGVHD in the Haplo HCT in our study (13%) was similar to that previously reported with PTCy [10, 21, 22], but higher than that reported by Wang et al. with ATG-based prophylaxis (6%) [7]. This may be explained by differences in variables such as age, conditioning regimens and stem cell source, among others. Interestingly, we observed no significant differences in the cumulative incidence of grade II–IV and III–IV aGVHD in ALL according to donor type, as observed in previous studies [4].

The present study did not find statistically significant differences in extensive cGVHD between the three cohorts in univariate analysis. However, the low incidence of this in all cohorts, but especially in the Haplo cohort (11%), deserves to be noted. As recently demonstrated by the ALWP-EBMT, the addition of immunosuppressive drugs to PTCy enhances its effect and reduces the risk of severe cGVHD, reducing mortality and improving survival [23, 24]. The influence of stem cell source was also relevant, as almost 50% of the patients in the Haplo group received BM. The use of BM seems to lower the risk of cGVHD in haplo setting [25]. In addition, recent studies have observed lower GVHD in patients receiving BM haploidentical transplants compared to PB MUD transplants [26].

In contrast to what was observed in patients with AML in CR1 transplanted under a similar prophylaxis of GVHD, in whom a significantly higher rate of NRM and a lower rate of relapse were found in the haploidentical setting compared to MSD and MUD [26], the present study in patients with ALL in CR1 could not detect any difference in these outcomes between the three groups. The lack of significant differences in NRM and relapse translated into an absence of significant differences between the three cohorts not only in OS and LFS, but also in GRFS. All these outcomes are in line with those reported in other studies analyzing patients with ALL in CR1 [5, 7, 8]. In the two comparative studies, as in ours, there were also no statistically differences in NRM, relapse, OS, LFS, and GRFS between alloHCT from haploidentical donor compared with MSD using ATG [26] or with MUD using PTCy or ATG [5]. In a subgroup analysis of patients in CR1 from another comparative study that included patients in all stages of the disease, no differences were also observed for these outcomes when the haploidentical receptors and MUD were compared [4].

The 2-year relapse incidence of 20% in the haploidentical cohort is in line with that reported in other studies analyzing patients with ALL in CR1 [7, 8]. The PTCy strategy seems to offer a good balance between GVHD prevention and antileukemic efficacy. Interestingly, a recent ALWP-EBMT study comparing PTCy with ATG in haploidentical HCT for ALL showed reduced relapse risk with PTCy [10]. Relapse rate was particularly low in Ph + patients, for which excellent results using universal PTCy strategy have been recently described [27]. Although we did not have data on the use of tyrosine kinase inhibitors in this study, it is likely that their use pre- and post-transplant may have contributed to the low relapse rate.

We should emphasize that the use of TBI was the only factor identified in the multivariate analysis associated with a better LFS. Although the advantage of TBI over chemotherapy in ALL has never been prospectively analyzed, a few meta-analysis [28, 29] and retrospective studies [30, 31] have previously suggested this advantage of TBI in adults with ALL.

Due to the retrospective nature of a registry-based study, some potential bias cannot be ruled out. To avoid an important source of bias, the analysis was restricted to patients with ALL in CR1 and those using PTCy as GVHD prophylaxis. However, a variety of conditioning regimens were used and there were obvious differences in the stem cell source and GvHD prevention strategies, such as in vivo TCD or the addition of other immunosuppressive drugs, depending on the type of donor. In fact, patients undergoing HCT from MSD and MUD more frequently received PB and in vivo TCD than did Haplo transplant recipients. Although some of these factors could be adjusted for in multivariable analysis, cell sources, TCD, and combination of immunosuppressive drugs for GvHD prophylaxis were strongly associated with type of donor and their effect could not be evaluated. Therefore, the results of this study should be interpreted in the context of these different transplant packages that include donor type together with stem cell source, TCD and GVHD prophylaxis strategies. In addition, important variables such as pre-transplant minimal residual disease and comorbidity index were not available. Despite the heterogeneity in these factors, the analysis of a large series of patients limited to an early stage of a single disease (ALL in CR1) who used a homogeneous GvHD prophylaxis with PTCy, allowed us to segregate the effect of donor from the effect of GvHD prophylaxis. Future studies, like the ongoing European prospective randomized study comparing MUD to Haplo (Haplo-MUD Study) with PTCy in both arm (ClinicalTrials.gov Identifier: NCT04232241), will be able to overcome some of these limitations.

## Conclusions

In patients with ALL undergoing allo-SCT, PTCy for GVHD prophylaxis seems promising and should be compared prospectively to standard regimens in order to establish the standard of care. In this scenario, under homogeneous GVHD prophylaxis, transplant outcomes with different donor types were similar, providing evidence to support Haplo transplantation as a valuable alternative donor option in this setting.

## Data Availability

The dataset supporting the conclusions of this article are available in the ALWP of EBMT in Paris, Saint Antoine Hospital.

## Abbreviations

aGVHD: Acute GVHD
allo-SCT: Allogeneic stem cell transplantation
ALWP: Acute Leukemia Working Party
ALL: Acute lymphoblastic leukemia
BM: Bone marrow
cGVHD: Chronic GVHD
CR1: First complete remission
EBMT: European Society for Blood and Marrow Transplantation
GRFS: GVHD-free and relapse-free survival
GVHD: Graft-versus-host
Haplo: Haploidentical donors
HLA: Human leukocyte antigen
LFS: Leukemia-free survival
MAC: Myeloablative conditioning
MSD: Matched sibling donors
MUD: Matched unrelated
NRM: Nonrelapse mortality
PB: Peripheral blood
PTCy: Post-transplant cyclophosphamide
RIC: Reduced intensity conditioning
SCT: Stem cell transplantation
OS: Overall survival
